# Necroptosis and autophagy in cisplatinum-triggered nephrotoxicity: Novel insights regarding their prognostic and diagnostic potential

**DOI:** 10.1016/j.toxrep.2024.101807

**Published:** 2024-11-12

**Authors:** Mai O. Kadry, Rehab M. Abdel-Megeed

**Affiliations:** National Research Center, Therapeutic chemistry deparment, Al Buhouth Street, Cairo, Egypt

**Keywords:** Cisplatin, Nephrotoxicity, *XBP*, *CHOP*, *C-Myc*

## Abstract

Necroptosis is an innovative class of programmed autophagy (Atg) and necrosis; considered as a type of homeostatic housekeeping machinery that have observed an escalating concern due to its power in alleviating Cisplatinum-induced nephrotoxicity. This article elucidated in details the prospective role of both autophagy and necroptosis on Cisplatinum-triggered nephrotoxicity and investigating more potent therapy via lactoferrin and Ti-NPS conjugation. Cisplatinum is a commonly used chemotherapeutic drug; one of the limiting adverse actions of cisplatinum is renal toxicity. Upon cisplatinum administration, autophagy is highly stimulated in the kidney to shield against nephrotoxicity. Atg is a lysosomal degradation process which discards detorirated proteins to retain cell homeostasis. This article summarizes necroptosis progress in reconizing cisplatinum nephrotoxicity and debates how this progress can help in discovering more potent therapy via lactoferrin and Ti-NPS conjugation via monitoring autophagy and apoptotic biomarkers X-box-binding protein 1 *(XBP),* C/EBP homologous protein *(CHOP),* hypoxanthine phosphoribosyltransferase-1 *(HPRT),* FKBP prolyl isomerase 1B *(FKBP),* Cellular myelocytomatosis oncogene *(C-myc),* tumor suppressor gene *(P53)* and tumor necrosis factor *(TNF-α)*. Cisplatinum nephrotoxicity was conducted in rat model via an oral dose of (2 mg/kg BW) for one month furthermore a comparative study was conducted among TiNPs-loaded Cisplatinum and Lactoferrin loaded Cisplatinum. Loaded drug delivery system counteracted Cisplatinum triggered nephrotoxicity via controlling autophagy and apoptotic *XBP, CHOP, HPRT, FKBP, C-myc,* P53 and TNF-α signaling pathway.

## Highlights

• Induction of cisplatinum nephrotoxicity in animal model.
• Treatment with TiNPs-loaded Cisplatinum and Lactoferrin loaded Cisplatinum.
• Monitoring autophagy biomarkers *XBP and CHOP.*
• Monitoring inflammatory and apoptotic biomarkers *HPRT/FKBP/C-myc/* P53/TNF-α.
• Monitoring DNA damage and morphological changes.

## Graphical Abstract

**Figure:**
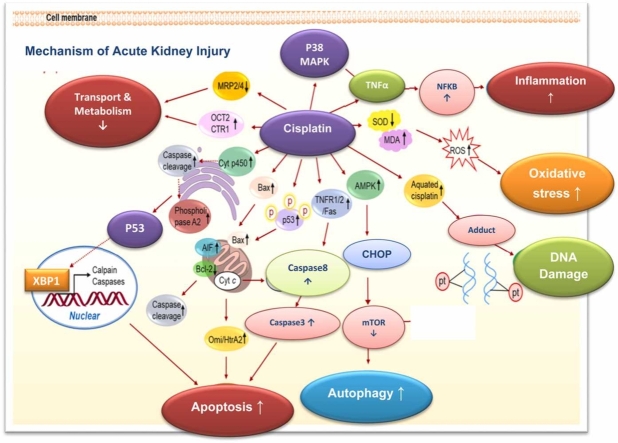

## 1 Introduction

Necroptosis hallmark shares the features of programmed (apoptosis) and random (necrosis) that is characterized morphologically by cellular swelling, plasma membrane rupture and organelle malfunction. Energy depletion, generation of ROS and Ca2+ are other biochemical characteristics of necroptosis [31]. Necroptosis dysregulation has been linked to cancer, drug-induced nephrotoxicity and ischemia/reperfusion injury [30], [32], [43]. The necroptotic signaling pathway is regulated by a cascade of kinases as receptor-interacting protein kinase (RIPK)-1 and RIPK3 [52], [57]. Metabolic disturbance, anticancer agents, and promotion of death receptors (TNFR & TLRs) can all triggers necroptosis and autophagy [14]. In spite of several stress accountable for necroptosis, TNF-α signaling pathway still the well known promotor of necroptosis [52]. TNFα binding to TNFR1 receptor can result in cell apoptosis, or necroptosis. Triggering TNFR1 causes morphological variation in the receptor, allowing its cytoplasmic part to use various proteins to generate prosurvival complex I (RIPK1, TNFR-associated death domain and TNFR-associated factors II and V) [27], [36], [50]. Dephosphylation of RIPK1 causes the formation of complex II, that includes caspase-8 and RIPK1 which cleaves (RIPK3 & RIPK1) thus activates apoptosis. On the other hand, once caspase-8 is hindered, RIPK1 links to RIPK3 to create the necrosome complex, which is then phosphorylated and forms mixed lineage kinase domain-like protein oligomers that disturp osmotic homeostasis. Main machineries of necroptosis can either inhibit or trigger macroautophagy. RIPK3 and RIPK1 trigger the stimulation of AMPK which enhances the motivation of macroautophagy via AMPK/TSC/mTORC1 signaling pathway. RIPK3 phosphorylates ULK1 to simplify alternative macroautophagy. It was well-established that the crosslink among necroptosis and autophagy is mediated via the structural and functional interaction among Beclin-1, CHOP and the anti-apoptotic biomarkers Bcl-XL and Bcl2. Autophagy (Atg) is a lysosomal degradation process that discards cytoplasmic malfunction ingredients. Under normal physiological conditions, Atg possess a vital function in conserving kidney homeostasis, regulate kidney renovation and renal necrosis [29]. The most predominant kind of Atg is macroautophagy, in which the cell generates a double-membrane isolated compartment called phagophore that develops into autophagosome [44]. Under CIS toxicity, Atg is triggered in response to cellular stress [10], [9], [23]. Thus, activation of Atg can sheild the renal tissue versus acute CIS damage, nevertheless its impact on renal cancer is dose-dependent [Xiaoru et al., 2021]. CIS-nephrotoxicity includes different stimuli and signaling pathways as endoplasmic reticulum stress (ERS), DNA and mitochondrial damage causing renal tubular cell damage and necrosis Sun et al., 2019; [35], [37]. ERS biomarkers XBP1, CHOP and GRP78 were elevated in CIS-induced nephrotoxicity. Furthermore, increased ER stress was linked to severe CIS induced renal damage; renal tubular cell apoptosis and kidney necrosis [Huang et al., 2020]. Cisplatinum is a powerful treatment for solid tumors but it is toxic to various organs particularly the kidney [11]. Acute kidney injury (AKI) arises in nearly 30–40 % of subjects who receive cisplatinum and is categorized by impairment in renal function, increase in nitrogen metabolism end products, tubule dilatation and tubular cell necrosis and death. According to Brillet et al. and Latcha et al., CIS may cause subclinical but permenant reduced glomerular filtration rate. CIS nephrotoxicity is a complex process that includes various pathophysiological processes, including microvascular problems, ERS, tubular cell apoptosis and inflammatory interaction. The proximal tubule, particularly the S3 segment, is highly suspective and prone to injury in AKI models [67]. CIS is mostly taken up by renal tubular cells via copper transporter 1 (CTR1) and organic cation transporters 2 (OCT2) then pass through a number of bio-activation procedures that produce hazardous metabolites, that are catalyzed by -glutamyl transpeptidase (GGT) and cysteine-S-conjugate –lyase [19], [18]. However, it can bind DNA, creating inter- and intrastrand cross-links, DNA damage and DNA-damage response, ultimately leading to cell cycle arrest and apoptosis [13]. When exposed to CIS, multiple signaling pathways, including MAPKs and p53-DNA damage response signaling, are activated, resulting in renal tubular cell apoptosis, necroptosis, and ferroptosis via triggering PIDD and PUMA-α. CIS can cause OS and ER stress [60], and mitochondrial dysfunction as well [66]; Yan et al., 2016; [62]. Novel drug delivery systems were recently developed to bypass the obstacle of CIS nephrotoxicity among them TiO2-NPs loaded and lactofferin loaded CIS that can trigger sustained drug release; improve bioavilability and therapeutic index by reducing its nephrotoxicity. Lactoferrin provide a protective impact versus cisplatin-triggered renal toxicity via reducing renal CIS accumulation, inflammation and apoptosis. CIS can cause renal functional impairment and histopathological damage; it can also interrupt the redox balance in the kidney and triggers inflammatory response via STAT1 signaling. Conversly, Cis-loaded NPs had a minor stimulatory action on the inflammatory and apoptotic signalling cascades, via diminshed renal morphological and functional changes. Cis-loaded TiNPs may be a valuable potential medication for reducing CIS cytotoxicity and so preserving renal function via m-TOR, PI3K, caspase-3 and P53 modulation thus hinder renal apoptosis [4]. Our main target is confirming that ERS and necroptosis are hallmarkes in CIS-induced nephrotoxicity and that Lac-CIS and TiNPs-CIS designed formulations can prevent CIS- nephrotoxicity and lowers its consequence.

## 2 Materials and methods

### 2.1 Chemical supplementation

Cisplatinum, Lactoferrin and Titanium NPs were obtained from Pharmacia Company and Sigma-Aldrich Co (St. Louis, MO, USA) respectively. RT-PCR kits for detecting *C-Myc, HPRT, FKBP-5, XBP and CHOP* gene expression were obtained from Qiagen (USA).

### 2.2 Animals and treatments

Thirty two male Wistar Albino rats from the animal house of the National Research Center, weighing 180–200 g, were used. Housing was in controlled conditions of (50 % humidity, 20 °C, and a 12-hour cycle of darkness and light) and free access to normal chow diet and water. The Animal Care and Use Committee of the National Research Center and the US National Institute of Health approved the ethical practices and policies (19−293) that were rigorously followed in all procedures involving the treatment and care of animals.

### 2.3 Experimental design

Acclimatization for one week was followed by animal’s separation into four groups/8 rats.

G 1: Rats received saline (Normal group).

G 2: Rats recieved Cisplatinum orally (2 mg/kg BW) for one month [40].

G 3: Rats received Lactoferrin-loaded Cisplatinum orally (2 mg/kg BW) for one month [55].

G 4: Rats received Titanium loaded- Cisplatinum orally (2 mg/kg BW) for one month [56].

### 2.4 Blood sampling and tissue preparation

Rats were carbon dioxide sedated and sublingual vein blood was collected, sera were centrifuged at 4000 rpm for 15 min. Animals were euthanized by cervical dislocation and kidney tissue was separated and homogenized in phosphate buffer with a pH of 7.4 (20 % w/v) and utilized for biochemical testing.

### 2.5 Measured biochemical parameters

#### 2.5.1 Serum creatinine

Detected spectrophotometry via Randox Company kits in accordance to manufactures guidelines [7].

#### 2.5.2 Serum urea

Urea was estimated spectrophotometry via Randox Company kits in accordance to manufactures guidelines [7], [2].

#### 2.5.3 mRNA gene expression of kidney CHOP, XBP, HPRT, FKBP5 and C-myc

RT- PCR detects CHOP, XBP, HPRT, FKBP5 and C-myc via specific primers (Table 1). Total RNA was first extracted from kidney tissue and converted to cDNA, then further, amplified via RT-PCR kits. The thermal profile: 50°C for 3 min, 92°C for 10 min, 92°C for 40–57°C for 30 s, 70°C for 32 s, and 75°C for 15 min [25], [1].

**Table 1:** Primers sequence designed for RT-PCR gene expression.

Primers	Sequence
Hprt1	Forward: 5′-GCTTCCTTCTCCGCAGACT−3′ Reverse: 5′-CTTCATCACGTCTCGAGCAA−3′
c-Myc	Forward: 5′-ATCACAGCCCTCACTCAC−3′ Reverse: 5′-ACAGATTCCACAAGGTGC−3′
FKBP−5	Forward: 5′-GAACCCAATGCTGAGCTTATG−3′ Reverse: 5′-ATGTACTTGCCTCCCTTGAAG−3′
CHOP	5'-ATGGCAGCTGAGTCATTGCCTTTC−3′ 5'-AGAAGCAGGGTCAAGAGTGGTGAA−3′
XBP	5'-CCGCAGCAGGTGCAGG−3′ 5'-GAGTCAATACCGCCAGAATCC−3′
β-actin	Forward: 5′-CTTTGATGTCACGCACGATTTC−3′ Reverse: 5′-GGGCCGCTCTAGGCACCAA−3′

### 2.6 Determination of P53 and TNF-α by ELISA assay

The P53 and TNF-α protein expression were determined using ELISA kits (R&D systems, MN, USA). The microplate was pre-coated with specific antibodies. Further, the immobilized AB that binds to P53 and TNF-α was supplemented, and then secondary AB specific for P53 and TNF-α was added. Then measured at 450 nm [24], [26].

### 2.7 Statistical analysis

Results were accessible as mean ± SEM. Statistical analysis utilized GraphPad Instat 3 and SPSS 16 via one-way ANOVA followed by POST-Hoc and Tukey’s test. *P*-value of <0.05 statistically significant.

## 3 Results

### 3.1 Modulation of HPRT and C-myc mRNA gene expression

**Fig. 1:**
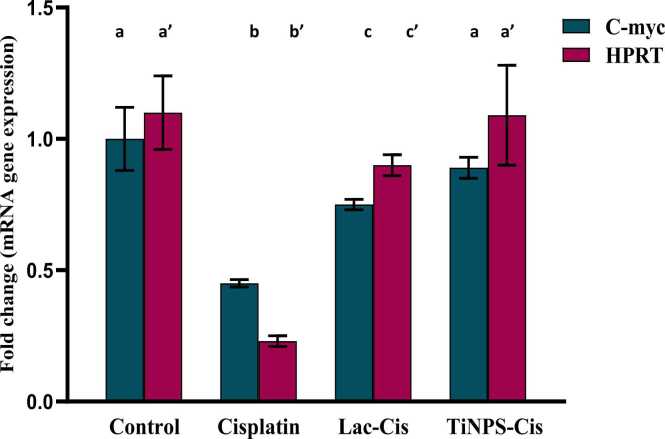
Impact of Lactoferrin loaded- Cisplatimum and Titanium loaded- Cisplatimum on kidney HPRT and C-Myc gene expression post Cisplatimum nephrotoxicity. Data are expressed as means ±SEM (n = 8), P<0.05. Groups having different letters are considered significantly different, while, groups having similar letters are not significantly different from each other. Β-actin was used as refrence gene.

Fig. 1 deduced that CIS intoxication revealed a significant down-regulation in mRNA gene expression of C-Myc and HPRT and with 0.4&0.2 fold changes respectively, in comparison to the control value. Nonetheless, up-regulation was significant in those groups cured with Lactoferrin loaded-CIS with 0.7&0.9 fold changes respectively, and Titanium loaded-CIS with 0.9&1.2 fold changes respectively, highlighting TiNPs-CIS regimen superiority in preventing CIS-induced nephrotoxicity and resistance.

### 3.2 Modulation of renal mRNA gene expression of FKBP5

**Fig. 2:**
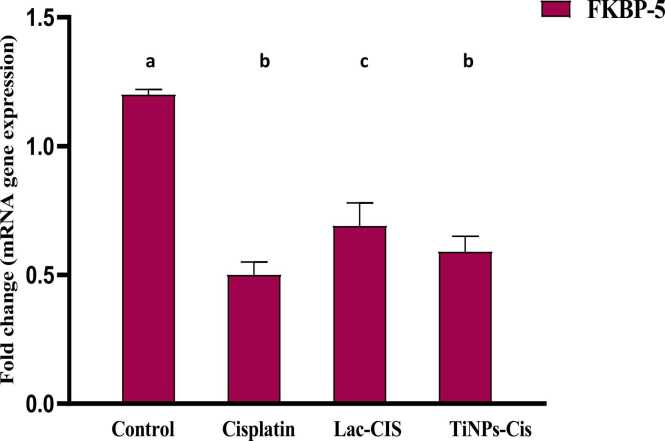
Impact of Lactoferrin loaded- Cisplatimum and Titanium loaded- Cisplatimum on kidney FKBP-5 gene expression post Cisplatimum nephrotoxicity. Data are expressed as means ±SEM (n = 8), P<0.05. Groups having different letters are considered significantly different, while, groups having similar letters are not significantly different from each other. Β-actin was used as refrence gene.

Data in Fig. 2 deduced that CIS intoxication revealed a significant down-regulation in the gene expression of FKBP with 0.5 fold change in comparison with the normal. Nonetheless, up-regulation was significant in Lactoferrin loaded-CIS and Titanium loaded-CIS with 0.75&0.6 fold changes respectively, reflecting Lac-CIS regimen superiority.

### 3.3 Modulation of renal mRNA gene expression of CHOP and XBP

**Fig. 3:**
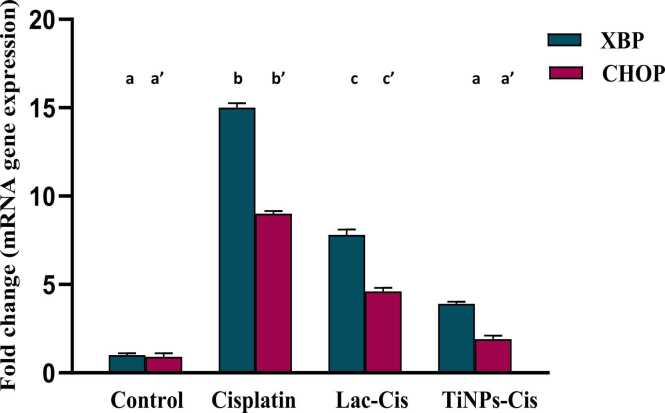
Impact of Lactoferrin loaded- Cisplatimum and Titanium loaded- Cisplatimum on kidney XBP and CHOP gene expression post Cisplatimum nephrotoxicity. Data are expressed as means ±SEM (n = 8), P<0.05. Groups having different letters are considered significantly different, while, groups having similar letters are not significantly different from each other. Β-actin was used as refrence gene.

Fig. 3 deduced that CIS intoxication caused a significant up-regulation in the gene expression of XBP and CHOP with 15&9 fold changes respectively, in comparison to the normal value. Nonetheless, down-regulation was significant in Lactoferrin loaded-CIS with 7&5 fold changes respectively, and Titanium loaded-CIS with 4&2 fold changes respectively, reflecting, TiNPs-CIS regimen superiority in repressing autophagy.

### 3.4 Modulation of renal TNF-α and P53 protein expression

**Fig. 4:**
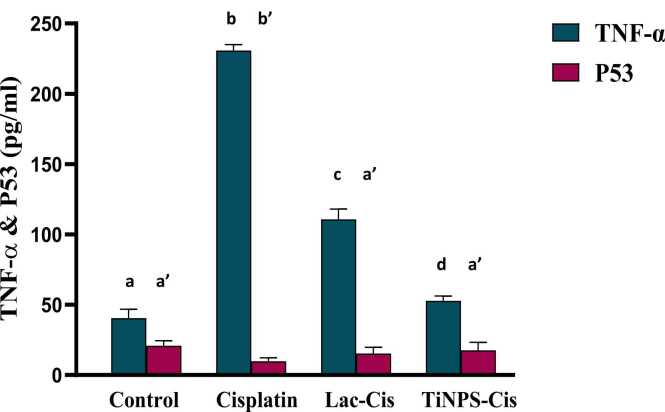
Impact of Lactoferrin loaded- Cisplatimum and Titanium loaded- Cisplatimum on kidney TNF-α and P53 prorein expression post Cisplatimum nephrotoxicity. Data are expressed as means ±SEM (n = 8), P<0.05. Groups having different letters are considered significantly different, while, groups having similar letters are not significantly different from each other.

Fig. 4, illusterated that CIS intoxication caused a marked increase in the inflammatory biomarker TNF-α protein expression with a mean value of 220 (pg/ml) and a marked decrease in apoptotic biomarker P53 with a mean value of 10 (pg/ml) as compared to the normal value. Nonetheless, amelioration in TNF-α was significant post Lactoferrin loaded-CIS treatment with a mean value of 110 (pg/ml) and Titanium loaded-CIS treatment with a mean value of 50 (pg/ml) with the superiority of TiNPs-CIS regimen as compared with CIS group. On the other hand, elevation in P53 was significant post Lactoferrin loaded-CIS treatment with a mean value of 20 (pg/ml) and Titanium loaded-CIS treatment with a mean value of 30 (pg/ml) with the superiority of TiNPs-CIS regimen in reducing inflammation and renal apoptosis.

### 3.5 Inhibition of Cisplatinum -Induced renotoxicity

**Fig. 5:**
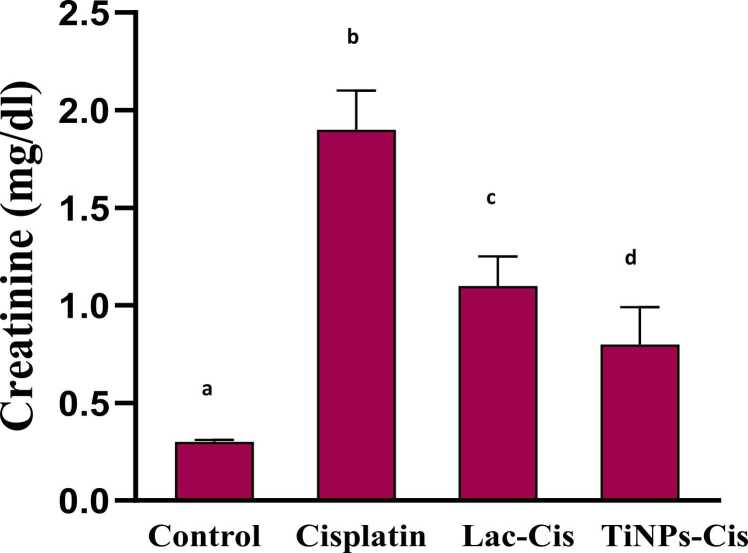
Impact of Lactoferrin loaded- Cisplatimum and Titanium loaded- Cisplatimum on serum creatinine post Cisplatimum nephrotoxicity. Data are expressed as means ±SEM (n = 8), P<0.05. Groups having different letters are considered significantly different, while, groups having similar letters are not significantly different from each other.

**Fig. 6:**
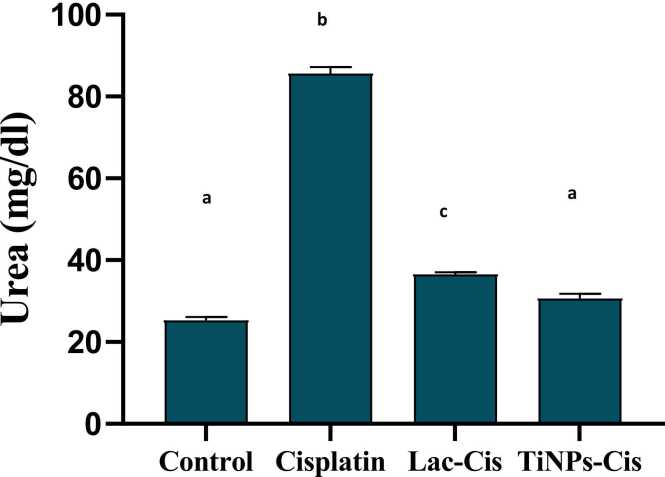
Impact of Lactoferrin loaded- Cisplatimum and Titanium loaded- Cisplatimum on serum urea post Cisplatimum nephrotoxicity. Data are expressed as means ±SEM (n = 8), P<0.05. Groups having different letters are considered significantly different, while, groups having similar letters are not significantly different from each other.

Fig. 5, Fig. 6, CIS intoxication increased significantly serum creatinine and urea levels with mean values of 1.9 & 82 (mg/dl) respectively, as compared with the normal. Conversely, in Lactoferrin loaded-CIS and Titanium loaded-CIS, the levels of kidney biomarkers were relatively lower with a mean value of 1& 0.8 (mg/dl) respectively, for creatinine and with a mean value of 35& 30 (mg/dl) respectively,for urea with TiNPs-CIS revealing the most significant impact in improving kidney function.

**Fig. 7:**
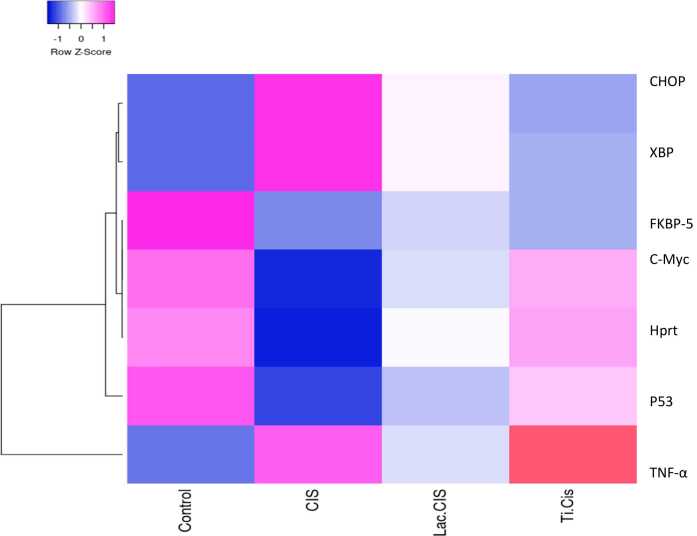
Heatmap representing different gene expression; blue represents low score however red represent high score.

## 4 Discussion

Necroptosis is activated via multiple exogenous and endogenous stresses, comprising metabolic disorder, anticancer drugs and stimulation of death receptors as TLRs and TNFR. Binding of TNF-α to TNFR1 results in the formation of complex I, stabilizing complex I leads to cell survival and inflammation via activating MAPK and NFƙ-B pathways. PGAM5 phosphorylates cyclophilin D once activated converses phosphorylation of dynamin-related protein 1, leading to stimulation and leads to mitochondrial division and opens transition pore, finally, contributing to necroptosis [34], [33].

The present study elucidated that post CIS intoxication; autophagy biomarkers (CHOP and XBP) were notecibily elevated in addition to the inflammatory biomarker TNF-α. Meanwhile, Ti-CIS and Lact-CIS treatments amileorated these altered parameters, with TiNPs-CIS revealing the highest impact. This results coincide with that once TNF-α oligomers translocates into the endoplasmic reticulum, they trigger a stress reaction leading to intracellular Ca2+ uptake, resulting in necroptosis through damaging cell membrane, stimulating ROS, that triggers mPTP opening, and further promoting Ca2+-dependent enzymes as phospholipase and calpains contributing to lysosomal membrane permeability and consequent secreation of cathepsin D& B into the cytoplasm promoting oxidative potential and Lysosomal dysregulation, resulting in necroptosis [14]. Regardless of various stimuli accountable for necroptosis origination, TNF-α still the most likely important necroptosis trigger [52]. TNF-α binding to its receptor (TNFR1) can results in apoptosis or cell survival or necroptosis (Seifert and Miller, 2017; [14]. Alternatively, when complex-I was disturbed or the ubiquitination procedure was repressed, RIPK1 detaches from it and combines with caspase-8 and Fas-associated death domain, creating apoptotic complex II and inducing TNF-induced apoptosis [50]. Apoptosis was the major contributor of CIS nephrotoxicity. Necroptosis and oxidative stress explain how the prooxidant CIS, could trigger tubular necroptosis. CIS increases the generation of ROS as H_2_O_2_, OH^●^, and O_2_^●^ via stimulating NADP-oxidase enzymes [16]. Furthermore, CIS impairs mitochondrial function by hindering the stimulation of different antioxidant enzymes as GST, GPX and SOD, leading to an imbalance among oxidant/antioxidant defense mechanism, resulting in OS [28]. CIS can disturb the mitochondrial respiratory chain, contributing to ROS generation and mitochondrial malfunction [38]. Furthermore, during the process of converting to a highly powerful nephrotoxic metabolite, CIS combine to GSH depleting its antioxidant activity [38]. ROS promotes RIPK1 autophosphorylation, allowing RIPK3 recruitment and necrosome generation [15]. Cisplatinum is an antineoplastic medication that treat malignancies of the kidney, lung and breast. Nevertheless, nephrotoxicity, myelosuppression and ototoxicity are possible adverse effects of Cisplatinum [17].

The current study elucidated that following CIS adminstiration, renal function biomarkers (creatinine and urea) were significantly increased. Meanwhile, Ti-CIS and Lact-CIS treatments altered these elevated parameters, with TiNPs-CIS reflecting the highest influence. CIS nephrotoxicity in experimental animals showed histopathologic alterations of acute tubular necrosis associated with azotemia. The development of renal insufficiency often occurs several days after the Cisplatinum administration, accompained with increase in urea and creatinine. Cisplatinum is eliminated via the renal tissue by glomerular filtration as well as tubular secretion. Cisplatinum accumlation in the renal tissue surpass the blood, indicating drug concenteration in kidney parenchymal cells [63]. According to previous animal studies, Cisplatinum experiences metabolic stimulation in the renal tissue, resulting in a highly powerful toxin. The synthesis of glutathione conjugates in the circulation, possibly triggered via GST, is the first step in this process. GSH-conjugates are split into cysteinyl-glycine-conjugates as they move through the kidney via GGT, which is represented on the membrane of the proximal tubules [65]. Aminodipeptidases that exist on the membrane of proximal tubules, metabolize cysteinyl-glycine-conjugates into cysteine-conjugates that are converted to active thiols by cysteine-S-conjugate-β-lyase [Zhang et al., 2003] leading to mitochondrial malfunction and depletion in mitochondrial antioxidant defense mechanisms [6].

Herein, post CIS intoxication, the tumor suppressor P53 gene expression was significantly reduced. In the meantime, this altered gene was reversed post Ti-CIS and Lact-CIS treatments, with TiNPs-CIS superiority. Cisplatinum molecules that have not been bound are filtered and highly transported, by proximal tubular epithelial cells. Its nephrotoxicity is mediated by ROS, endoplasmic reticulum stress pathway, inflammation and extrinsic and intrinsic apoptotic pathways and fibrogenesis. These elements cause tube destruction, K^+^ and Na^+^ malfunction, and Mg deficiency. TNF-α orchestrated the inflammatory process by binding to TNFR1 and TNFR2 receptors and creating two complexes. Complex 1 stimulates the transcription of NF-kB and numerous inflammatory and cell survival genes, whereas Complex-II triggers the stimulation of caspase-10 and −8, contributing to apoptosis stimulation. TNF is generally unnoticeable in healthy kidneys, however most renal cells create it in response to stimuli such as Cisplatinum. [42]. Once renal epithelial cells are exposed to Cisplatinum, Bax is translocated to the mitochondria, activating caspase 2 and cytochome c and further caspase 9 is activated [58]. Both p53-dependent caspase 6 and 7 expression [61] and p53-independent caspase activation via Bax/Bak-induced cytochrome-C contributes to CIS-triggered tubular epithelial cell apoptosis and necroptosis [21]. DNA adduct formation is thought to promote Cisplatinum cytotoxicity, mutagenicity and tumorigenicity. Cisplatinum makes covalent connections with the purine bases in DNA, leading to intrastrand 1,2- or 1,3-cross-linking. Cisplatinum binding to DNA inhibits gene transcription and DNA replication and may contribute to double strand breakage. The resulting genotoxic stress activates a signaling cascade, resulting in p53 phosphorylation and activation and further cell cycle arrest or apoptosis [8], [10], [9]. P53 has emerged as a key modulator of Cisplatinum-triggered apoptosis. As a result of DNA mutation and oncogene stimulation, p53 promotes cell cycle arrest or cell death. Genetic suppression of p53 transcriptional action ameliorated cisplatinum-triggered caspase stimulation and apoptosis in vitro and cisplatin-triggered cell death and kidney damage in vivo [22]. Two p53 transcriptional targets, p53-induced protein with a death domain (PIDD) and p53-regulated modulator of apoptosis-alpha (PUMA-α), may facilitate p53 effects in cisplatin cell death [58]. Cisplatinum activates p53, that further promotes PIDD inturn caspase 2, resulting in mitochondrial necroptosis [51]. Cisplatinum activation of p53 may be caused by DNA damage and oxidative stress [20]; Basnakian et al.;[64].

Herein, post CIS intoxication, the resistance biomarkers genes *HPRT* and *C-myc* were significantly reduced. In the meantime, these altered genes were reversed post Ti-CIS and Lact-CIS treatments, with TiNPs-CIS revealing the most significant impact. Cisplatinum's mutagenicity in the Hprt gene of CHO-K1 cells was previously examined. CIS induced dose-related elevation in mutant consequence. The mutation spectrum of cisplatin was G:C-->T:A transversion, cisplatin produced tandem base-pair substitutions, primarily at positions 135/136, and a greater frequency of G:C-->A:T transition [39]. Combined Hprt and Kim-1 reporter genes reproducibly determined CIS-promoted nephrotoxicity in S3 cells (Kukora et al., 2016). MYC expression is typically upregulated in malignancies. According to a recent study of over 9000 human tumors, MYC gene amplification occurs in around 28 % of cases [49]. Cisplatinum elevates both c-Myc and cyclin E in the course of inducing a DNA damage-resistance phenotype and combined therapy of palbociclib and the c-Myc bromodomain inhibitor JQ1 has a synergistic apoptotic impact on Cisplatinum-resistant cells [46].

FKBP51 (tacrolimus-binding protein 51) regulates proteins included in various processes, as cell development, cancer, immunity, inflammation, cell plasticity and differentiation [48], [59]. Herein, post CIS intoxication, FKBP51 gene expression was significantly down regulated. In the meantime, this altered gene was modulated post Ti-CIS and Lact-CIS treatments, with Lac-CIS revealing the most significant impact. Agrowing evidence revealed that immunophilin FKBP51 is elevated in different cancer kinds, with a major rise in leucocytes of oxaliplatin chemotherapy patients. Data revealed that FKBP51 has a role in Cisplatinum toxicity in kidney tubules [45]. FKBP51 participates in steroid receptor pathways by transporting from the cytoplasm to the mitochondria or nucleus, there it controls Akt, NFƙ-B, TGF-α and TNF-α pathways [Zgajnar et al., 2019]. FKBP51 is highly promoted in tumors [54] and participates in cancer cell antiapoptotic mechanisms [47]. FKBP51 shield from Cisplatinum inflammatory response, as reflected via TNF-α. FKBP51 down regulation participate in TNFα/NF-κB inflammatory cascade [5]. FKBP51 hinders nuclear transport of the p50_RelA/p65 complex, impairing the transcriptional impact of NF-κB [Erlejman et al., 2014] thus inhibiting inflammation [53]. Cisplatinum elevated FKBP51 expression, implying that early antiapoptotic action controlled via FKBP51 is at least partly responsible for apoptosis or necroptosis [41].

Cisplatinum, an effective cancer treatment, frequently causes nephrotoxicity, limiting its therapeutic efficiency and applicability. Cis-NC effectively produced from irradiation chitosan coated Cisplatinum and MgO-NPs to trigger sustained Cisplatinum release and improve therapeutic efficacy by reducing nephrotoxicity. Cisplatinum caused renal functional impairment and histopathological damage; it also interrupted the redox balance in the kidney and triggered inflammatory response via STAT1 signaling. Furthermore, Cisplatinum-triggered mTOR stimulation and PI3K/Akt signalling inactivation were combined with elevation of p53 and caspase3 to produce renal apoptosis. Cis-NC had a minor stimulatory action on the inflammatory and apoptotic signalling cascades, via undetectable renal morphological and functional changes. Cis-NC may be a valuable potential medication for reducing Cisplatinum cytotoxicity and so preserving renal function and structure [4].

AgNPs-CIS have been shown to reduce mitochondrial apoptosis. Furthermore, NPs might reduce Cisplatinum-induced dephosphorylation of AKT, phosphorylated p38, MAPK and phosphorylated JNK. Nanocurcumin has the potential to reduce oxidative stress by serving as a ROS scavenger [3]. CONPs have the potential to inhibit LPOO and inflammatory biomarkers. Furthermore, exosomes protected the kidney from Cisplatinum-induced damage by decreasing Bcl2 and elevating Bax, cleaved caspase-9, and −3. As a result, NPs- drug delivery methods for Cisplatinum are so promising that lipoplatins and NPS, have reached phase 1–3 studies [12] by modifying the biocomptability of certain targeted medicines in cancer therapy enhancing in vivo efficacy and reducing side effects is the most relevant impact (Lu et al., 2023; Zhuang et al., 2022].

## 5 Conclusion

A comparative study between cisplatinum, TiNPs-Cisplatinum and Lac-Cisplatinum revealed a promising candidates countracting Cisplatinum nephrotoxicity via TiNPs and lactoferrin conjugation and modulating XBP, CHOP, HPRT, FKBP, TNF, P53 and C-Myc signaling pathways.

## Ethics approval and consent to participate

Ethics number is (19−293) in National Research Center.

## Funding

This work has no financial support.

## Author statement

The corresponding author is responsible for ensuring that the descriptions are accurate and agreed and reviewed by all authors
The role(s) of all authors:
Mai O Kadry: Designed the experiment, biochemical analysis, statistical analysis, write and submitted the manuscript.
Rehab M Abdel-Megeed: Biochemical analysis and statistical analysis.
No funder is present for this work.

## Declaration of Competing Interest

The authors declare that they have no known competing financial interests or personal relationships that could have appeared to influence the work reported in this paper.

## Data Availability

No data was used for the research described in the article.
